# Human Pentraxin 3 Binds to the Complement Regulator C4b-Binding Protein

**DOI:** 10.1371/journal.pone.0023991

**Published:** 2011-08-22

**Authors:** Anne Braunschweig, Mihály Józsi

**Affiliations:** Junior Research Group Cellular Immunobiology, Leibniz Institute for Natural Product Research and Infection Biology – Hans Knöll Institute, Jena, Germany; French National Centre for Scientific Research, France

## Abstract

The long pentraxin 3 (PTX3) is a soluble recognition molecule with multiple functions including innate immune defense against certain microbes and the clearance of apoptotic cells. PTX3 interacts with recognition molecules of the classical and lectin complement pathways and thus initiates complement activation. In addition, binding of PTX3 to the alternative complement pathway regulator factor H was shown. Here, we show that PTX3 binds to the classical and lectin pathway regulator C4b-binding protein (C4BP). A PTX3-binding site was identified within short consensus repeats 1–3 of the C4BP α-chain. PTX3 did not interfere with the cofactor activity of C4BP in the fluid phase and C4BP maintained its complement regulatory activity when bound to PTX3 on surfaces. While C4BP and factor H did not compete for PTX3 binding, the interaction of C4BP with PTX3 was inhibited by C1q and by L-ficolin. PTX3 bound to human fibroblast- and endothelial cell-derived extracellular matrices and recruited functionally active C4BP to these surfaces. Whereas PTX3 enhanced the activation of the classical/lectin pathway and caused enhanced C3 deposition on extracellular matrix, deposition of terminal pathway components and the generation of the inflammatory mediator C5a were not increased. Furthermore, PTX3 enhanced the binding of C4BP to late apoptotic cells, which resulted in an increased rate of inactivation of cell surface bound C4b and a reduction in the deposition of C5b-9. Thus, in addition to complement activators, PTX3 interacts with complement inhibitors including C4BP. This balanced interaction on extracellular matrix and on apoptotic cells may prevent excessive local complement activation that would otherwise lead to inflammation and host tissue damage.

## Introduction

The long pentraxin 3 (PTX3) is a soluble recognition molecule of the innate immune system that belongs to the family of pentraxins [1], [2]. PTX3 shares the typical pentraxin domain with the short pentraxins C-reactive protein (CRP) and serum amyloid P, and has an additional unique N-terminal domain. In contrast to CRP and serum amyloid P, which are mainly produced in the liver and are distributed systemically, PTX3 is produced locally by several cell types including endothelial cells, fibroblasts and immune cells, such as monocytes, macrophages, myeloid dendritic cells and neutrophil granulocytes [3]. The expression and release of PTX3 are enhanced upon inflammation and infection, leading to significantly increased PTX3 plasma levels (200–800 ng/ml versus <2 ng/ml in normal plasma) under these conditions [4].

PTX3 is a glycoprotein that forms elongated, asymmetric octamers with a molecular mass of approximately 340 kDa [5], [6]. PTX3 binds to several host ligands, such as complement components, the extracellular matrix (ECM) proteins TSG-6 and inter-α-trypsin inhibitor, P-selectin and apoptotic cells [7], [8]. Moreover, PTX3 recognizes certain pathogens including fungi (e.g., *Aspergillus fumigatus*), gram-positive and gram-negative bacteria (e.g., *Streptococcus pneumoniae*, *Pseudomonas aeruginosa*), and viruses. Studies with *Ptx3^−/−^* mice have shown that PTX3 plays an essential role *in vivo* in the resistance against selected pathogens, in particular *Aspergillus fumigatus* [9].

PTX3 can mediate phagocytosis of recognized targets via Fc-receptors on phagocytes, a mechanism shared with the short pentraxins [10], [11]. In addition, PTX3 enhances target opsonization by interaction with the complement system [12], [13]. Complement is a powerful innate effector system that is aimed at recognizing and eliminating microorganisms and participates in the safe removal of apoptotic and damaged host cells [14]. During these processes, complement closely collaborates with other innate recognition systems such as pentraxins and Toll-like receptors [15], [16]. The complement system can be activated via three pathways, namely the classical, lectin and alternative pathway, which could lead to target opsonization, lysis and inflammation. As overwhelming and misdirected complement activation is potentially destructive to human tissues, complement activation is restricted in the host by several membrane-bound and soluble inhibitors.

The short pentraxin CRP was shown to bind C1q and L-ficolin and thereby to activate the classical and lectin complement pathways [17], [18]. In addition, CRP interacts with the complement inhibitors factor H (CFH) and C4b-binding protein (C4BP) [19], [20]. CFH is the major soluble regulator of the alternative complement pathway [21], whereas C4BP is the main regulator of the classical and lectin pathways in plasma [22]. The simultaneous binding of both, complement activatory (C1q, L-ficolin) and inhibitory (CFH, C4BP) proteins likely ensures an optimal opsonization of target surfaces without the detrimental effects of overwhelming complement activation [23].

C4BP is a 570-kDa, multimeric glycoprotein with a concentration of approximately 200 µg/ml in plasma [22]. The major C4BP isoform is composed of seven α-chains and one β-chain, which contain 8 and 3 short consensus repeat (SCR) domains, respectively. C4BP inhibits complement activation by acting as a cofactor for factor I in the cleavage and inactivation of C4b, and by its decay accelerating activity for the classical pathway convertases [22]. In plasma, C4BP is circulating in complex with the vitamin K-dependent anticoagulant protein S, which mediates the binding of C4BP to negatively charged phospholipid membranes and to dead cells [24].

PTX3, similar to CRP, binds C1q and L-ficolin, as well as mannan-binding lectin (MBL) and M-ficolin, and modulates the classical and lectin complement pathways and thus opsonization [12], [13], [25], [26]. Recently, PTX3 was shown to bind to CFH [27]. Here, we identify C4BP as a novel PTX3 binding protein, and demonstrate functionally relevant interactions of PTX3 with this classical/lectin complement pathway inhibitor on ECM and on apoptotic cells.

## Materials and Methods

### Ethics statement

This study has been approved by the ethics committee of the Medical Faculty of Friedrich Schiller University Jena (control number 2268-04/08). All healthy blood donors provided written informed consent.

### Materials

Recombinant human PTX3 (carrier protein-free, purity >95% as assessed by SDS-PAGE/silver staining, endotoxin level <1 EU/µg PTX3), MBL, L-ficolin, biotinylated goat anti-human PTX3 antibody and mouse anti-human PTX3 monoclonal antibody (mAb) were purchased from R&D Systems (Wiesbaden-Nordenstadt, Germany). Human CFH, factor I, C3b, C4b, C1q and goat anti-human C3 antiserum (recognizing C3b, iC3b and C3c) were obtained from Complement Technology, Inc (Tyler, TX, USA). C2-depleted serum, CRP, goat anti-human C1q antibody, goat anti-human C4 antibody, rabbit anti-human C5b-9 antibody, rabbit anti-human C4BP antiserum and horseradish peroxidase (HRP)-conjugated streptavidin were purchased from Merck (Darmstadt, Germany). Anti-C4c and anti-C4d mAbs were from Quidel (TECOMedical, Bünde, Germany). Purified human C4BP (purity >95%, with protein S <0.1%) was purchased from Hyphen BioMed (Neuville-sur-Oise, France). Recombinant SCR1–3 and SCR5–7 of the C4BP α-chain as Ig fusion proteins were kindly provided by Dr. Santiago Rodríguez de Córdoba (Madrid). Protein S and mouse anti-human protein S antibody were obtained from Haematologic Technologies (Essex Junction, VT, USA). HRP-conjugated rabbit anti-goat IgG, swine anti-rabbit IgG and rabbit anti-mouse IgG were obtained from DakoCytomation (Hamburg, Germany). Denatured, monomeric CRP (mCRP) was generated as described by Kresl et al. [28]. Normal human plasma (NHP) was collected from blood donors after informed consent.

### Dot blot analysis

To identify C4BP binding to PTX3, serial dilutions (200 – 3.125 ng) of PTX3, or BSA and C4b, used as negative and positive controls, respectively, were dotted onto nitrocellulose membrane. Non-specific binding sites were blocked in 5% dry milk in PBS (140 mM NaCl, 2.7 mM KCl, 10 mM Na_2_HPO_4_, 1.8 mM KH_2_PO_4_) containing 0.05% Tween-20 overnight at 4°C. C4BP (2 µg/ml) was added in Tris-buffered saline (TBS; 10 mM Tris, 140 mM NaCl, 2 mM CaCl_2_, 1 mM MgCl_2_, pH 7.4) containing 2% dry milk and 0.05% Tween-20 for 4 h at 20°C, and binding of C4BP was detected using C4BP antiserum and HRP-conjugated anti-rabbit Ig. In reverse experiments, C4BP, BSA and C1q (100 – 1.56 ng) were immobilized on the nitrocellulose membrane. After blocking, the membrane was incubated with PTX3 (5 µg/ml) in TBS as described above. Binding of PTX3 was detected using biotinylated anti-PTX3 antibody and HRP-conjugated streptavidin. The blots were developed using a chemiluminescent detection kit for HRP (Applichem, Darmstadt, Germany).

### Microtiter plate binding assays

Costar microtiter plates (Corning, NY, USA) were coated with C4BP (10 µg/ml) diluted in TBS overnight at 4°C. The wells were washed after each step with TBS containing 0.05% Tween-20. After blocking with 4% dry milk in TBS for 2 h at 37°C, PTX3 (0.625–10 µg/ml) diluted in TBS was added for 1 h at 37°C. Bound PTX3 was detected with a biotinylated anti-PTX3 antibody followed by HRP-conjugated streptavidin. TMB PLUS substrate (Kem-En-Tec Diagnostics, Denmark) was used to visualize binding and the absorbance was measured at 450 nm. In reverse experiments, PTX3 (5 µg/ml) was immobilized and serial dilutions of C4BP were added, followed by detection of bound C4BP using C4BP antiserum and the respective secondary antibody. Binding of the recombinant SCR1–3 and SCR5–7 fragments (20 µg/ml) of the α-chain to immobilized PTX3 was measured using the C4BP antiserum. To analyze the calcium-dependence of PTX3 binding to C4BP, the binding assay was performed in DPBS (Lonza, Wuppertal, Germany) containing 1 mM Ca^2+^ and 0.5 mM Mg^2+^ or DPBS without divalent cations and containing 10 mM EGTA.

In competition assays, PTX3 was preincubated with C1q, MBL, L-ficolin, CFH or protein S for 1 h at 20°C, then added to the C4BP-coated microtiter plates. To analyze the effect of C4BP ligands, C3b, C4b or mCRP was added together with PTX3 to immobilized C4BP. For inhibition assays, immobilized C4BP was preincubated with the respective antibody for 1 h at 20°C before incubation with PTX3.

To analyze binding of C4BP to PTX3 from plasma, serial dilutions of NHP or C2-depleted serum were added to PTX3- or gelatin-coated wells for 1 h at 37°C. Bound C4BP was detected using C4BP antiserum.

To determine the apparent dissociation constant, serial dilutions of PTX3 were incubated on C4BP-coated wells. Bound PTX3 was calculated by converting the OD values to nanomolar concentration using a standard curve for PTX3 and a molecular mass of 340 kDa for the recombinant PTX3 octamer [6]. The *K*_d_ was determined from non-linear regression analysis of the binding curve using SigmaPlot version 10.0.

### Cofactor assays for C4b inactivation

The influence of PTX3 on the cofactor activity of C4BP in the factor I-mediated cleavage of C4b in the fluid phase was analyzed by incubating factor I (5 µg/ml), C4b (5 µg/ml) and C4BP (25 µg/ml), preincubated with or without 25 µg/ml PTX3 for 30 min at 20°C, for 15 min at 37°C. In solid phase cofactor assays, 25 µg/ml C4BP was incubated on wells coated with 10 µg/ml PTX3, protein S or BSA for 1 h at 37°C, followed by the addition of 5 µg/ml factor I and 5 µg/ml C4b for 30 min at 37°C. After incubation, samples were subjected to SDS-PAGE under reducing conditions. Western blotting was used to detect C4b cleavage products using anti-C4 antibody.

### Extracellular matrix assays

To study the binding of PTX3 to human ECM, fibroblast cell-culture derived human ECM (MaxGel™; Sigma-Aldrich, Taufkirchen, Germany) diluted 1∶50 in TBS was immobilized on microtiter plate wells overnight at 4°C and used for subsequent binding assays (as described above). ECM of endothelial cells was prepared by culturing HUVEC (ATCC; LGC Promochem, Wesel, Germany) on gelatin-coated 96-well tissue culture plates (0.2% gelatin) in DMEM medium supplemented with 10% FCS, 1% L-glutamine and 50 µg/ml gentamicin sulfate for 7 days at 37°C in a humidified atmosphere containing 5% CO_2_. Cells were washed and released from the surface by incubation in DPBS containing 10 mM EDTA at 37°C. The cell-free ECM was washed with TBS and used immediately for subsequent binding assays (as described above). The production of ECM by endothelial cells was monitored by detecting ECM components after cell detachment, using antibodies against laminin, collagen type IV, and von Willebrand Factor. To analyze the influence of PTX3 on complement activation on ECM, PTX3 (10 µg/ml) was added to MaxGel™-coated wells for 1 h at 37°C, followed by incubation with 1.5% NHP for 40 min at 37°C in gelatin veronal buffer containing 0.15 mM Ca^2+^ and 0.5 mM Mg^2+^ (GVB; Sigma-Aldrich). C1q binding was measured using anti-C1q antibody. Deposition of C3 fragments and terminal pathway components was measured using polyclonal anti-C3 and anti-C5b-9 antibodies, respectively. C5a in the supernatants was measured by ELISA (Quidel; TECOMedical GmbH, Bünde, Germany).

### Interaction of PTX3 with C4BP on apoptotic cells

Jurkat cells (DSMZ, Braunschweig, Germany) were cultured in RPMI medium supplemented with 10% FCS, 1% L-glutamine and 50 µg/ml gentamicin sulfate at 37°C in a humidified atmosphere containing 5% CO_2_. Apoptosis was induced by 0.5 µM staurosporin (Sigma-Aldrich) for 24 h. Apoptosis was monitored by double staining with Annexin V-APC (Immunotools, Friesoythe, Germany) and DAPI. Cells positive for Annexin V staining but negative for DAPI staining were considered early apoptotic cells, whereas double positive cells were characterized as late apoptotic cells.

To analyze binding of PTX3, apoptotic cells were incubated with increasing concentrations of PTX3 in TBS containing 1% BSA for 30 min at 20°C. After washing, polyclonal anti-PTX3 antibody was added for 20 min at 20°C followed by FITC-labeled streptavidin (eBioscience). In some assays, cells were preincubated with PTX3 (15 µg/ml) and subsequently incubated with C4BP (2.5 µg/ml) for 30 min at 20°C. Binding of the complement regulator was detected using anti-C4BP antibody and the corresponding Alexa Fluor 488-labeled secondary antibody (Invitrogen, Darmstadt, Germany). Deposition of C3 fragments, C4c, C4d and C5b-9 were detected after incubation with 0.5% human plasma in GVB containing 1% BSA for 1 h at 37°C using the corresponding antibodies. Cells were measured using a BD LSRII (BD Biosciences, Heidelberg, Germany) flow cytometer. Data were analyzed using FACSDiva (BD Biosciences) and FlowJo softwares (Tree Star, Ashland, OR).

Cofactor activity of C4BP on apoptotic cells was also detected by Western blot. Apoptotic cells were incubated with 2.5 µg/ml C4BP with or without a preincubation with 15 µg/ml PTX3. After washing, C4b and factor I were added to the cells and C4b cleavage was detected in the supernatant as described above.

### Statistical analysis

Statistical analysis was performed using Student's *t*-test. A *p* value<0.05 was considered statistically significant.

## Results

### PTX3 binds to the classical and lectin complement pathway regulator C4BP

**Figure 1:**
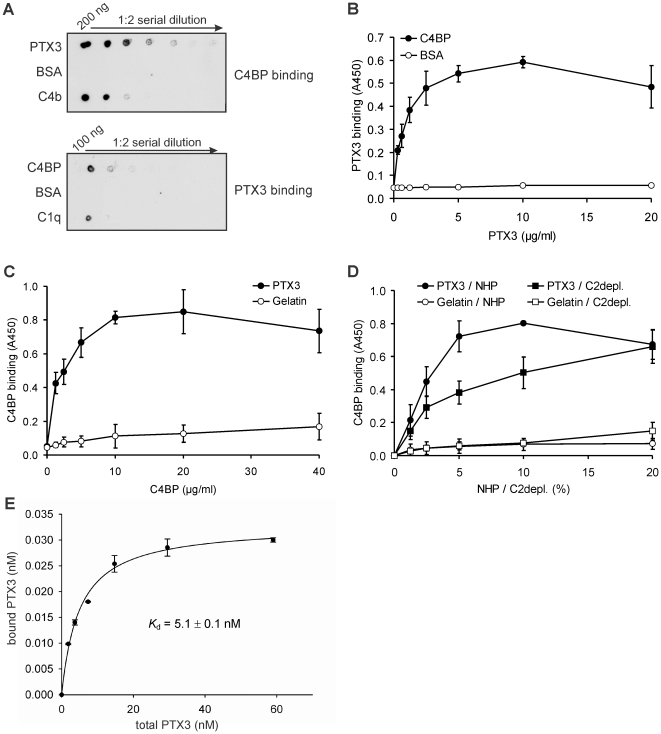
PTX3 interacts with the classical and lectin complement pathway regulator C4BP. (A) Dot blot analysis of PTX3-C4BP interaction. Serial dilutions of PTX3, BSA and C4b were applied on a nitrocellulose membrane, and binding of 2 µg/ml C4BP was detected using a C4BP antiserum (upper panel). Serial dilutions of C4BP, BSA and C1q were applied on a separate membrane, and PTX3 binding (5 µg/ml) was detected using a polyclonal anti-PTX3 antibody (lower panel). The blots are representative of two and three experiments, respectively. (B) Dose-dependent binding of recombinant PTX3 to immobilized C4BP or BSA, used as negative control, was measured by ELISA. (C) In reverse experiments, binding of C4BP to immobilized PTX3 or gelatin, used as negative control, was analyzed. (D) Immobilized PTX3 (black symbols) or gelatin (white symbols) was incubated with the indicated concentrations of normal human plasma (NHP; circles) or C2-depleted serum (C2depl.; squares). Binding of native C4BP was detected using a C4BP antiserum. In *B*, *C* and *D*, the data are means ± SD derived from three experiments. (E) Addition of increasing amounts of PTX3 results in a saturable binding to immobilized C4BP. Specific binding was measured using a standard curve of PTX3, and the *K*_d_ of 5.1±0.1 nM (mean ± SD from three experiments) was determined from non-linear regression analysis of the binding curve.

In order to analyze whether PTX3 is able to interact with the classical/lectin pathway regulator C4BP, first a dot blot assay was performed. Human PTX3, and BSA as negative control and C4b as positive control were dotted on a membrane in serial dilutions, then incubated with purified C4BP. C4BP binding was detected using a specific antiserum. This analysis showed a binding of C4BP to PTX3 (**Fig. 1A**). In a parallel assay, C4BP, BSA and C1q were applied on a membrane in serial dilutions, and the membrane was incubated with recombinant human PTX3. PTX3 bound to C4BP and to the positive control protein C1q (**Fig. 1A**). The binding of recombinant PTX3 to immobilized C4BP was also measured by ELISA. PTX3 bound to immobilized C4BP in a dose-dependent and saturable manner (**Fig. 1B**). In reverse experiments, a dose-dependent and saturable binding of purified C4BP to immobilized PTX3 was observed (**Fig. 1C**). We have also measured the binding of C4BP using increasing concentrations of human plasma (1–20%, containing ∼2–40 µg/ml C4BP) as a physiological source of C4BP. A saturable binding similar to that obtained with purified C4BP was found (**Fig. 1D**). Similarly, C4BP binding to PTX3 was detected from C2-depleted serum, excluding a role of complement activation fragments deposited on PTX3-coated wells in this interaction (**Fig. 1D**). To characterize the PTX3-C4BP binding, serial dilutions of PTX3 were incubated on immobilized C4BP, and bound PTX3 was calculated using a standard curve of PTX3. The calculated apparent dissociation constant (*K*_d_) was 5.1±0.1 nM, indicative of a high affinity interaction (**Fig. 1E**).

### The interaction of PTX3 and C4BP is not mediated by protein S

**Figure 2:**
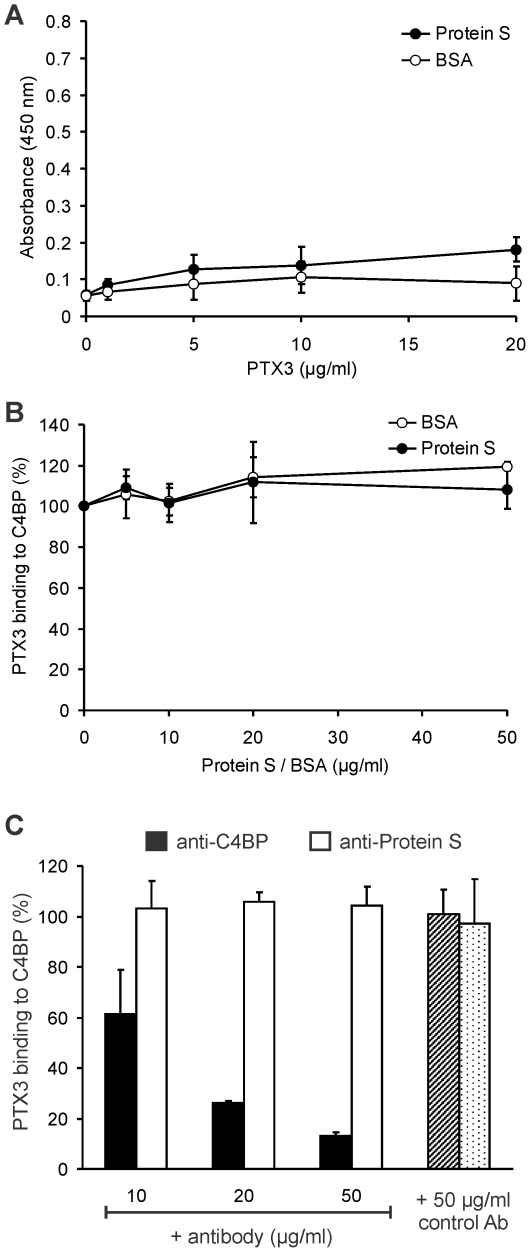
The interaction of PTX3 and C4BP is not mediated by protein S. (A) Binding of PTX3 to immobilized purified protein S and to BSA was measured by ELISA. No significant PTX3 binding to protein S was detected. The data represent means ± SD derived from three independent experiments. (B) Binding of PTX3 to C4BP was measured in the presence of increasing concentrations of protein S or BSA. The normalized values are means ± SD of data derived from three experiments. (C) Binding of PTX3 to immobilized C4BP was determined in the presence of the indicated concentrations of anti-C4BP (black bars), anti-protein S (white bars) and control antibodies (patterned bars). The normalized values are means + SD of data derived from three experiments.

Because most of C4BP is found in complex with protein S in plasma [24], we analyzed the influence of protein S on the C4BP-PTX3 interaction. PTX3 did not bind to immobilized, purified protein S (**Fig. 2A**) and *vice versa* (not shown). The binding of PTX3 to C4BP in the presence of increasing concentrations of protein S was also analyzed. Protein S did not show any effect on the PTX3-C4BP interaction (**Fig. 2B**). In addition, we used antibodies against protein S and C4BP in inhibition assays. The binding of PTX3 to C4BP was not influenced by an antibody against protein S, but it was inhibited by an anti-C4BP antibody (**Fig. 2C**). Altogether, these data indicate a specific direct interaction of C4BP and PTX3, in which protein S is not involved.

### The effect of calcium and known PTX3- and C4BP ligands on the interaction of PTX3 with C4BP

**Figure 3:**
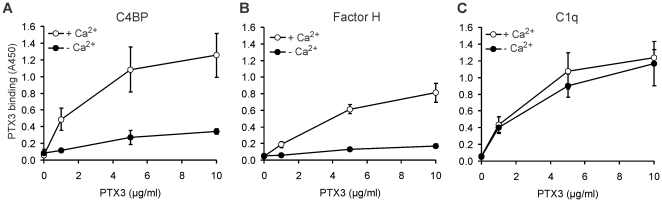
Influence of calcium on PTX3 binding to C4BP. Binding of recombinant PTX3 to immobilized (A) C4BP, (B) factor H and (C) C1q was determined in the absence (black circles) and in the presence (white circles) of 1 mM Ca^2+^ by ELISA. Data shown are means ± SD derived from three experiments.

PTX3 binds some of its ligands in a calcium-dependent manner. PTX3 binding to CFH was shown to be calcium-dependent, whereas its binding to C1q is calcium-independent [27]. In our assays, PTX3 binding to C4BP as well as to CFH, used as control, was strongly reduced in calcium-free buffer, whereas the C1q-PTX3 interaction was not influenced by the lack of calcium (**Fig. 3**).

**Figure 4:**
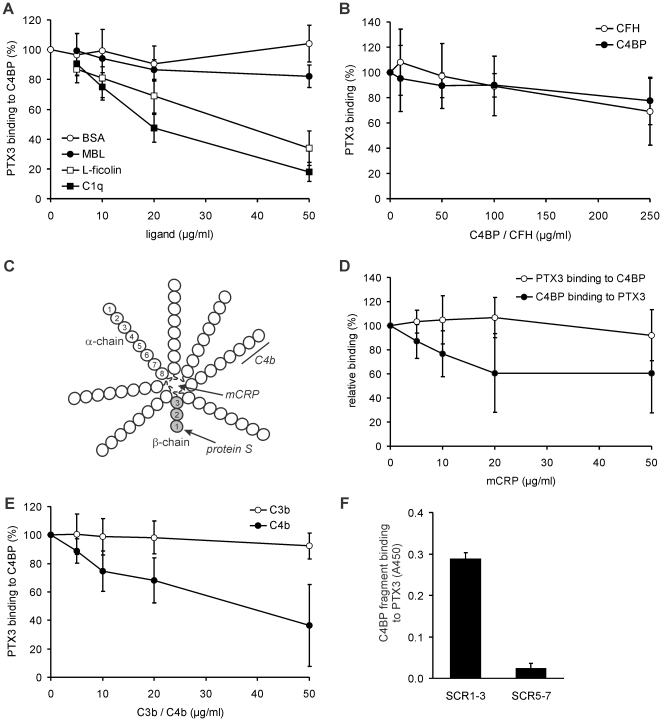
Influence of PTX3- and C4BP-ligands on the PTX3-C4BP interaction. (A) Binding of PTX3 to immobilized C4BP was measured in the presence of increasing concentrations of C1q, L-ficolin, mannan-binding lectin (MBL), or bovine serum albumin (BSA), used as control. (B) Binding of PTX3 to CFH was determined in the presence of increasing concentrations of C4BP, and that of C4BP in the presence of the indicated concentrations of CFH. No statistically significant competition between the two complement regulators for PTX3 binding was observed. (C) Schematic representation of C4BP. The most common C4BP isoform is composed of seven α-chains and one β-chain, each consisting of eight and three SCR domains, respectively. Protein S binds to C4BP via SCR1 of the β-chain. C3b binds within SCR1–4, and C4b binds to SCR1–3 of the α-chain, which is also responsible for the cofactor and decay accelerating activities of C4BP. mCRP binds to the central core of the C4BP molecule. (D) Binding of PTX3 to immobilized C4BP (white circles) and binding of C4BP to immobilized PTX3 (black circles) in the presence of increasing mCRP concentrations were measured by ELISA. (E) Binding of PTX3 to C4BP was determined in the presence of increasing concentrations of the C4BP ligands C3b and C4b. In *A*, *B*, *D*, *E*, the normalized values are means ± SD derived from three independent experiments. (F) Binding of recombinant SCR1–3 and SCR5–7 domains of the C4BP α-chain (20 µg/ml) to immobilized PTX3 was measured by ELISA. Data represent means + SD from three experiments.

To analyze whether C1q influences the interaction of PTX3 with C4BP, the binding of PTX3 to immobilized C4BP was measured in the presence of increasing C1q concentrations. C1q inhibited PTX3 binding to C4BP in a dose-dependent manner (**Fig. 4A**). Similarly, L-ficolin but not MBL inhibited the PTX3-C4BP interaction. In contrast to this, CFH even if applied in high concentrations, did not significantly inhibit PTX3 binding to C4BP and *vice versa* (**Fig. 4B**). Thus, complement regulators of both the alternative and the classical/lectin pathways can bind simultaneously to PTX3.

We also analyzed the effect of known C4BP ligands on PTX3 binding (**Fig. 4C**). C4BP binds via its central core to the short pentraxin CRP [20], preferentially to the denatured mCRP form [29]. We have generated mCRP by urea-chelation treatment, and measured PTX3 binding to C4BP using mCRP as competitor ligand. In these assays, mCRP up to 50 µg/ml concentration did not inhibit PTX3 binding. However, in reverse experiments the binding of C4BP to PTX3 was partially (∼40%) reduced by 50 µg/ml mCRP (**Fig. 4D**). The effect of the complement activation fragment C4b, which has a binding site in SCRs 1–3 of the C4BP α-chain, was also tested. Addition of C4b resulted in a partially reduced PTX3 binding by C4BP, whereas C3b, binding in SCRs 1–4, had no influence on this interaction (**Fig. 4E**). Altogether these data indicated a PTX3 binding site within SCR1–3 of the α-chain, and possibly a second binding site overlapping with that for mCRP. To further analyze binding sites, we used two fragments of the α-chain, covering domains SCR1–3 and SCR5–7. SCR1–3, but not SCR5–7, bound to immobilized PTX3 (**Fig. 4F**), confirming a PTX3 binding site in the N-terminal domains of the C4BP α-chain.

### PTX3-bound C4BP maintains its cofactor activity

**Figure 5:**
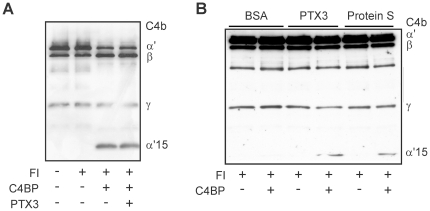
PTX3 does not influence the complement regulatory activities of C4BP. (A) Fluid phase C4BP cofactor assay. C4b was incubated with factor I (FI), C4BP and PTX3 in the indicated combinations. Samples were subjected to 10% SDS-PAGE under reducing conditions, transferred to nitrocellulose membrane, and C4b cleavage was detected by Western blot using a C4 antiserum. The C4b chains and the α′-chain cleavage product (α′15) are indicated on the right. One representative of three experiments is shown. (B) Solid phase C4BP cofactor assay. C4BP was added to wells coated with BSA, recombinant PTX3 or protein S. After washing, C4b and factor I were added at 37°C for 30 min, and C4b cleavage was analyzed from the supernatants by Western blot as described above. A representative experiment out of three is shown.

Next, we analyzed if the binding of PTX3 affects the complement regulatory function of C4BP. In a fluid-phase cofactor assay, the cofactor activity of C4BP for the factor I-mediated cleavage of C4b was measured. Identical C4b cleavage patterns were obtained in the absence and presence of PTX3 (**Fig. 5A**). In a solid-phase cofactor assay, i.e. when C4BP was bound to PTX3 on the microtiter plate surface, C4BP aided factor I in the inactivation of C4b (**Fig. 5B**). The C4b cleavage pattern and cleavage efficiency were similar to that observed with protein S-bound C4BP (**Fig. 5B**). Thus, C4BP maintains its complement regulatory activity when interacting with PTX3.

### PTX3 binds to human fibroblast- and endothelial cell-derived ECM and recruits C4BP

**Figure 6 pone-0023991-g006:**
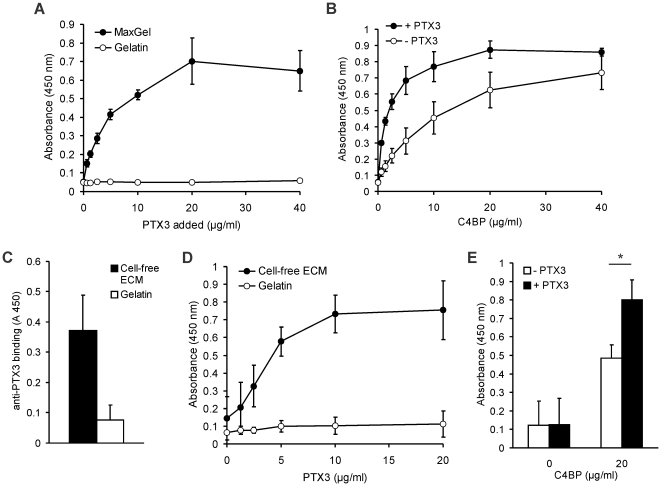
PTX3 recruits C4BP to human fibroblast- and to endothelial cell-derived ECM. (A) Dose-dependent binding of recombinant human PTX3 to microplate wells coated with human fibroblast-derived ECM (MaxGel™). Gelatin-coated wells served as control. Data shown are means ± SD from three experiments. (B) Dose-dependent binding of purified C4BP to MaxGel™ without or with preincubation with 20 µg/ml PTX3 was measured by ELISA. Data are mean values ± SD of three experiments. (C) PTX3 is present in HUVEC-derived ECM. HUVEC were cultured for 7 days in gelatin-coated 96-well plates, then the cells were detached, and PTX3 which was produced and released by HUVEC and bound to the ECM was detected using an anti-PTX3 mAb. Data shown represent mean + SD of three experiments. (D) HUVEC were cultured for 7 days in gelatin-coated 96-well plates, then the cells were detached and binding of PTX3 to the cell-free ECM was analyzed by ELISA. Gelatin-coated wells incubated with cell-culture medium were used as negative controls. Recombinant PTX3 was added in increasing concentrations and PTX3 binding was detected using a polyclonal anti-PTX3 antibody. Data shown represent mean ± SD of three experiments. (E) Binding of 20 µg/ml C4BP to HUVEC-derived ECM without (white bars) or with preincubation with 20 µg/ml PTX3 (black bars). PTX3 bound to the ECM significantly increased C4BP binding. Data shown are mean + SD derived from three independent experiments. * *p*<0.05, Student's *t*-test.

PTX3 and the complement regulator C4BP were shown to interact with certain components of the ECM [30]–[32]. Therefore, we set out to study the interaction of PTX3 with C4BP on ECM. To this end, we used the fibroblast-derived MaxGel™ as a model for human ECM. PTX3 and C4BP both bound to MaxGel™ in a dose-dependent manner (**Fig. 6A and 6B**). PTX3, when bound to the ECM, significantly enhanced the binding of C4BP (**Fig. 6B**). In contrast to this, C4BP did not influence the binding of PTX3 to the ECM (**not shown**). In addition, we have analyzed the interaction of PTX3 with C4BP on ECM produced by human endothelial cells *in vitro*. HUVEC were cultured in 96-well plates for 7 days, then the cells were removed by PBS containing 10 mM EDTA. The resulting cell-free ECM already contained detectable PTX3 produced by the endothelial cells (**Fig. 6C**). Exogenous PTX3 bound to HUVEC-ECM in a similar dose-dependent manner as observed for MaxGel™ (**Fig. 6D**). Furthermore, C4BP bound to HUVEC-ECM, similar to the binding on fibroblast-derived MaxGel™, and PTX3 significantly enhanced the binding of C4BP to endothelial cell-derived ECM (**Fig. 6E**). Thus, PTX3 binds to human ECM produced by fibroblasts or by endothelial cells, and recruits the complement regulator C4BP to these surfaces.

### The role of PTX3 and C4BP in complement activation on ECM

**Figure 7 pone-0023991-g007:**
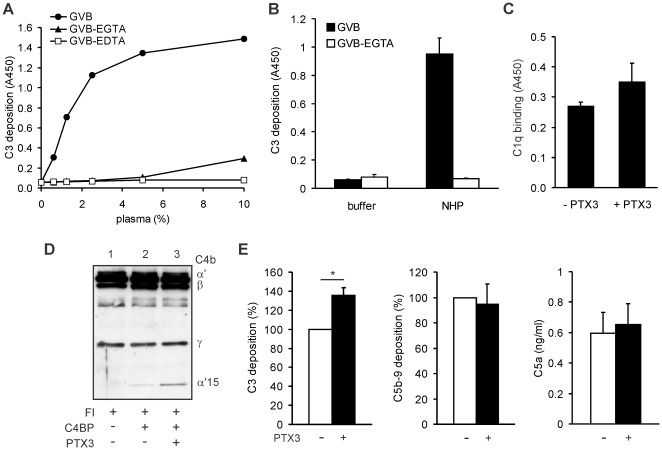
The role of PTX3 and C4BP in complement activation on ECM. (A) Microtiter plate wells coated with ECM (MaxGel™) were exposed to the indicated concentrations of human plasma, diluted in gelatin-veronal buffer (GVB; allowing activation of all complement pathways), in GVB-EGTA (allowing activation of the alternative pathway only), and in GVB-EDTA (no complement activation). C3 fragment deposition was measured by ELISA using an anti-C3 antibody. Data are means of values from two experiments. (B) The wells were coated with MaxGel™, preincubated with 20 µg/ml PTX3, then 1.5% NHP, or buffer only, was added in GVB and in GVB-EGTA. C3 fragments deposited to the wells were measured using an anti-C3 antibody. Data shown represent mean + SD of four experiments. (C) C1q binding from 1% NHP to wells coated with MaxGel and preincubated without or with 10 µg/ml PTX3 was measured using an anti-C1q antibody. Data represent mean + SD derived from three experiments. (D) Cofactor assay for C4b cleavage on ECM (MaxGel™). C4b and factor I (FI) were added to the ECM (lane 1, control) and to ECM preincubated with 20 µg/ml C4BP (lane 2) or first with 20 µg/ml PTX3 and then C4BP (lane 3). After incubation at 37°C for 2 h, the supernatants were separated on 10% SDS-PAGE and subjected to Western blotting. The blot was developed using a C4 antiserum. The C4b fragments are indicated on the right. One representative of three experiments is shown. (E) Complement activation on ECM was measured by adding 1.5% NHP to wells coated with MaxGel™ and preincubated without (white bars) and with 10 µg/ml PTX3 (black bars). Deposition of C3 fragments and of terminal pathway components was detected using anti-C3 and anti-C5b-9 antibody, respectively. The amounts of C5a in the supernatants were determined using a C5a ELISA kit. Data shown represent mean + SD of three experiments. * *p*<0.05, Student's *t*-test.

To analyze complement activation on ECM, wells coated with MaxGel™ were incubated with serial dilutions of human plasma in GVB buffer allowing activation of all pathways, in GVB containing 10 mM EGTA to detect alternative pathway activation only, and in GVB containing 10 mM EDTA to inhibit complement activation. Under our assay conditions, C3 deposition was observed in GVB only, indicating no direct activation of the alternative pathway on ECM (**Fig. 7A**). To assay the role of PTX3 on ECM, wells coated with MaxGel and preincubated with PTX3 were exposed to plasma. In GVB buffer allowing activation of all complement pathways, a strong C3 deposition was observed. When activation of only the alternative pathway was allowed in GVB-EGTA, no complement activation and C3 deposition was detected, indicating that PTX3 activates the classical/lectin pathway when bound on ECM (**Fig. 7B**). C1q binding was enhanced in the presence of PTX3, further confirming an enhanced classical pathway activation on ECM by PTX3 (**Fig. 7C**).

Next, the complement regulatory activity of C4BP on ECM was analyzed. When bound to ECM (MaxGel™), C4BP was still able to function as cofactor for the factor I-mediated cleavage of C4b (**lane 2 in** **Fig. 7D**). When the ECM was preincubated with PTX3, the increased binding of C4BP under these conditions (**Fig. 6B**) resulted in an enhanced inactivation rate of C4b (**lane 3 in** **Fig. 7D**).

Complement activation was compared in wells coated with MaxGel™ and preincubated with or without PTX3. C3 deposition was increased in PTX3-containing wells compared to wells with ECM only, whereas no enhanced deposition of terminal components (C5b-9) was observed on PTX3-containing wells (**Fig. 7E**). Similarly, the amount of C5a in the supernatants was not increased (**Fig. 7E**). Thus, although PTX3 enhances complement activation on ECM via C1q binding, this enhancement is limited to the early steps, and the activation beyond the C3 level is not increased due to the recruitment of fluid phase complement inhibitors, such as C4BP and CFH.

### The role of the PTX3-C4BP interaction on apoptotic cells

**Figure 8:**
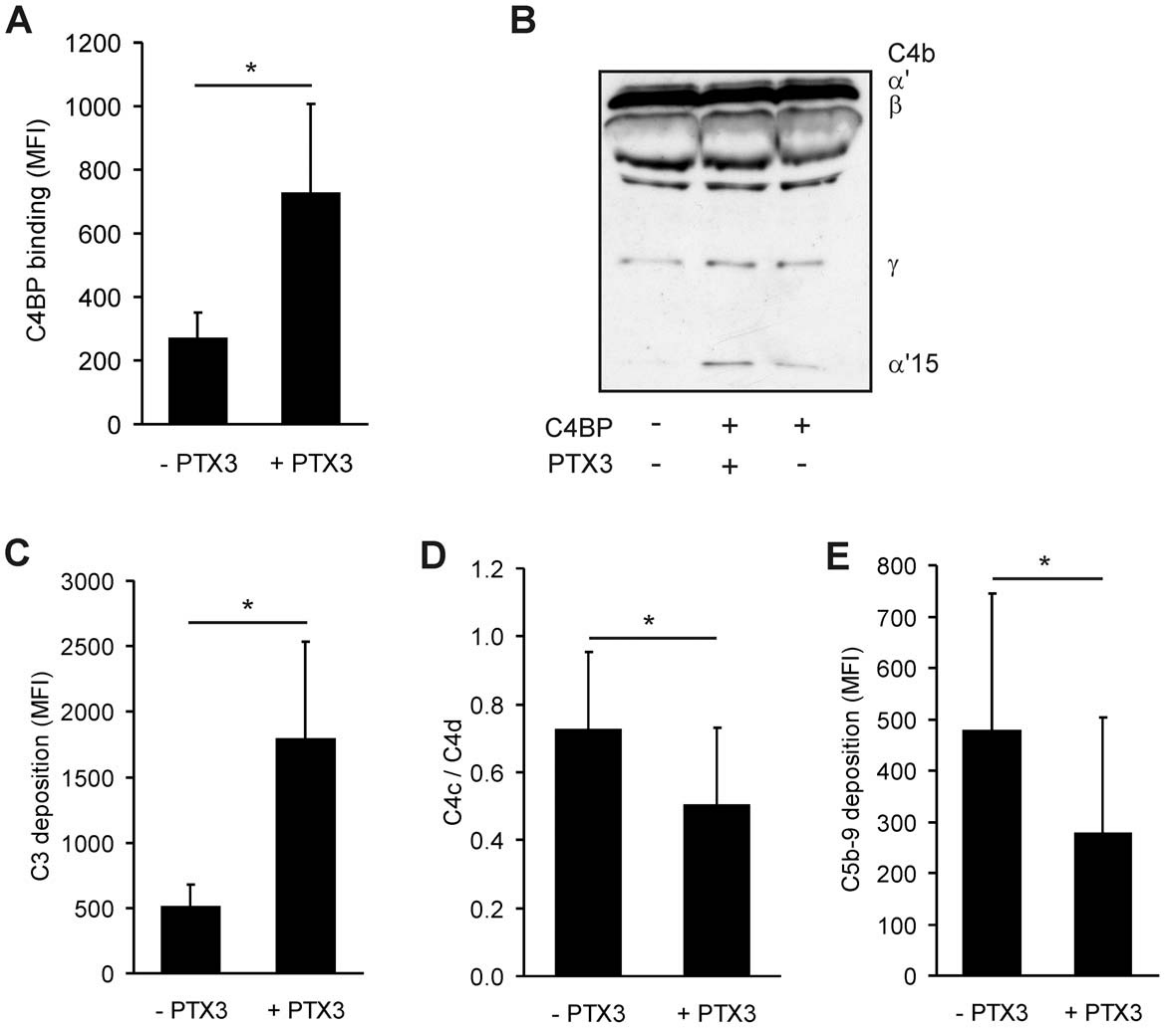
The role of the PTX3-C4BP interaction on apoptotic cells. (A) Binding of C4BP to late apoptotic Jurkat cells was measured without or with preincubation with PTX3 (15 µg/ml) by flow cytometry. Data show mean fluorescence intensity (MFI) values + SD from three independent experiments. Data without and with PTX3 preincubation were compared using paired *t*-test. * *p*<0.05. (B) Cofactor activity of C4BP bound on apoptotic cells. Apoptotic Jurkat cells were preincubated with PTX3 followed by the addition of C4BP. After washing, C4b and factor I were added, and C4b cleavage was detected from the supernatant by Western blot. A representative experiment out of three is shown. Apoptotic Jurkat cells preincubated without or with 10 µg/ml PTX3 were exposed to human plasma. (C) C3 deposition on the cells was measured by flow cytometry using a C3 antiserum that recognizes deposited C3b, iC3b and C3c fragments. Data represent means + SD of median fluorescence values (MFI) from five experiments. (D) The presence of deposited C4c and C4d fragments was detected by flow cytometry using anti-C4c and anti-C4d mAbs. The anti-C4c mAb only detects intact C4b fragments (because upon C4b cleavage the C4c fragment will not remain surface bound), and the anti-C4d mAb detects all deposited C4b, i.e. both intact and cleaved C4b, since the C4d-part remains cell-bound. Thus, a C4c/C4d = 1 indicates intact deposited C4b, whereas a C4c/C4d<1 indicates C4b cleavage. Data are expressed as the ratio of C4c- and C4d-specific median fluorescence values, and represent means + SD of calculated C4c/C4d ratios from three experiments. (E) C5b-9 deposition was detected by an anti-C5b-9 antibody. Data represent means + SD of median fluorescence values (MFI) from six experiments. * *p*<0.05, Student's *t*-test.

PTX3 binds to apoptotic cells and participates in the regulation of their safe clearance [15], [33]. Binding of the fluid phase complement regulators C4BP and CFH to opsonized apoptotic cells prevents excessive complement activation on the surface and lysis of the cells [34]. PTX3 was shown to enhance the binding of CFH to apoptotic cells [27]. Therefore, we investigated whether PTX3 is also able to recruit the classical/lectin pathway complement inhibitor C4BP to apoptotic cells. To this end, apoptotic Jurkat cells were generated and the binding of PTX3 as well as C4BP was assayed by flow cytometry. PTX3 bound in a dose-dependent manner to late apoptotic Jurkat cells, but not to early apoptotic and to viable cells (**not shown**), in agreement with previous reports [27], [33]. The binding of C4BP was significantly enhanced when PTX3 was present on the surface of late apoptotic cells (**Fig. 8A**), whereas we did not observe a recruitment of PTX3 by C4BP to apoptotic cells (not shown). The cell-bound C4BP was active as a cofactor for the degradation of C4b, and a preincubation of the cells with PTX3 resulted in an enhanced C4b cleavage (**Fig. 8B**). Complement activation on the cells was studied by exposing the apoptotic cells to human plasma in the absence and in the presence of PTX3. Preincubation of the cells with PTX3 caused an increased C3 fragment deposition (**Fig. 8C**), as also reported by Deban et al. [27], due to an enhanced initiation of the classical/lectin pathways. The presence of the various C4b fragments deposited on the cells was measured using anti-C4c and anti-C4d mAbs. While some of the C4b fragments were inactivated due to cell-bound C4BP in the absence of PTX3, as expected (**Fig. 8A and 8B**; [34]), the rate of C4b-inactivation on the apoptotic cell surface was significantly enhanced when preincubated with PTX3 (**Fig. 8D**). In parallel with this, the deposition of the lytic C5b-9 terminal complex was reduced when PTX3 was present on the cells (**Fig. 8E**). These data show that whereas PTX3 binding results in an enhanced complement activation on apoptotic cells, the simultaneously recruited C4BP enhances the rate of C4b inactivation. This C4BP activity contributes to an attenuation of the terminal pathway, acting in concert with CFH [27], [34]. Note that the alternative pathway will also be activated and CFH will promote C3b inactivation [27], [34]. Thus, besides activating complement and facilitating adequate opsonization [12], PTX3 can recruit regulators including C4BP that limit excessive complement activation on apoptotic cells.

## Discussion

In the present study we demonstrated a novel and functionally relevant interaction of PTX3 with C4BP, the major soluble regulator of the classical and lectin pathways, on biological surfaces such as the ECM and apoptotic cells.

In plasma, the main C4BP isoform is composed of seven α-chains and one β-chain, and forms a complex with protein S (bound via the β-chain), which mediates binding of this complex to apoptotic cells [24], [35]. The binding of C4BP to PTX3 was independent of protein S (**Fig. 2**), thus C4BP binds directly to PTX3 and can be recruited by this pentraxin to the surface of apoptotic cells (**Fig. 8**). Importantly, PTX3 did not interfere with the complement regulatory activity of C4BP, and C4BP was fully functional as a complement inhibitor when recruited by PTX3 to surfaces (**Fig. 5**–[Fig pone-0023991-g006] [Fig pone-0023991-g007] **8**). Thus, PTX3 is able to trigger a restricted activation of the classical complement pathway on target surfaces, i.e. the initial activatory step is enhanced, but the later steps of complement activation are not increased due to PTX3-bound complement regulators including C4BP. It is important to note, that the alternative pathway will also be activated secondary to classical/lectin pathway activation, due to the generation of C3 convertases, and enhanced via the amplification loop. Thus, and because of the binding of initiator molecules of complement to PTX3 (such as C1q), C3 deposition will be increased. An escalation of the cascade will however be prevented by the concerted action of membrane bound and recruited fluid phase regulators, such as C4BP and CFH [27], [34] (**Fig. 8**). This process can lead to an enhanced opsonization of targets, e.g. apoptotic cells, without the danger of runaway complement activation and complement-mediated inflammation.

We could identify a PTX3-binding site within SCR1–3 of the C4BP α-chain using recombinant deletion constructs (**Fig. 4**). The binding of C4BP to its complement ligands C4b and C3b is mediated via the SCRs 1–4 of the α-chain [22]. Addition of C4b but not C3b resulted in a partial inhibition of PTX3 binding by C4BP (**Fig. 4**), indicating a partially overlapping PTX3 binding site in the N-terminal SCRs of the α-chain, and confirming the results obtained with the recombinant SCR1–3 fragment. However, rather high amounts of C4b were required for a significant inhibitory effect; in line with this, PTX3-bound C4BP was still able to act as a cofactor for the inactivation of C4b (**Fig. 5**).

The short pentraxin CRP was shown to interact with C4BP, and this binding involves the central core of C4BP [20]. C4BP has a binding preference to modified or denatured CRP (mCRP) rather than to the naturally occurring pentameric CRP form [29], and native CRP when bound on apoptotic or necrotic cells does not enhance C4BP binding [34]. Although the *in vivo* existence and the biological relevance of mCRP are debated, we used it as a C4BP ligand with known binding site in a competition assay to help mapping the binding sites for the long pentraxin PTX3. When using mCRP as a competitor, there was only a partial inhibition of PTX3 binding to C4BP, indicating that besides SCR1–3 an additional PTX3 binding site that is partially overlapping with the mCRP site may be located at or near the central core of C4BP. The differing results from this assay performed in both direction, i.e. when C4BP or PTX3 were immobilized (**Fig. 4D**), might be attributed to the different availability of the C4BP central core and/or the different affinities of mCRP to fluid phase and surface bound C4BP.

The PTX3-ligands C1q and L-ficolin competed with C4BP for PTX3 binding, but C4BP and CFH did not significantly inhibit the binding of each other to PTX3 (**Fig. 4**). Thus, regulation of PTX3-induced complement activation by C4BP will include inactivation of C3/C5-convertase enzymes and C4b fragments, as well as competition with initiator molecules of complement. Similar to some other PTX3 ligands including CFH [27], the interaction of C4BP with PTX3 was influenced by calcium; the binding observed in the physiological presence of calcium was strongly reduced when calcium was absent from the buffer. The calculated apparent dissociation constant of 5.1 nM indicates a high affinity interaction between PTX3 and C4BP, and is similar in magnitude to those reported for PTX3-CFH and PTX3-C1q interactions (*K*_d_ = 110 nM and *K*_d_ = 74 nM, respectively; both were calculated considering PTX3 monomers) [7], [27].

PTX3 is an ancient and conserved pattern recognition molecule of the innate immune system that is important in protection against certain infections [9]. PTX3 binds to selected pathogens and can play a role in their opsonophagocytic removal directly and by generating further opsonins via complement activation [13], [36]. At the same time, the interaction of PTX3 with complement regulators that allow a certain degree of opsonization but block the escalation of the cascade, could prevent excessive and chronic inflammation which may be deleterious to the host. The interaction with complement regulators is even more important during host tissue injury and in the removal of apoptotic/necrotic cells in a non-inflammatory way [15], [23].

The binding of complement proteins (e.g., C1q and MBL) and pentraxins can enhance the uptake of apoptotic cells by phagocytes via specific receptors. Moreover, pentraxins can enhance C1q binding, which may lead to complement activation, the deposition of C3- and C4-derived opsonins [12], [15], [34], and potentially to activation of the terminal pathway. The expression of membrane-anchored complement inhibitory molecules is down-regulated on apoptotic cells [34], [37], which could cause an enhanced susceptibility of these cells to complement-mediated lysis. This loss in membrane-bound regulators can partially be compensated for by the binding of soluble regulators [34]. However, whereas the binding of CRP results in an increased C1q binding and complement deposition on apoptotic cells [34], [38], native CRP does not enhance C4BP and CFH binding [34]. In contrast to this, Deban et al. showed previously that PTX3 can increase the binding of CFH to apoptotic cells. When the cells were incubated with serum in the presence of PTX3, more C3 deposition occured, but C3 was quickly inactivated and no cell lysis was observed [27]. Here we show that the binding of C4BP to the surface of apoptotic cells is also enhanced by PTX3, which results in an enhanced C4BP regulatory activity on these cells. This contributes to preventing an uncontrolled escalation of the cascade (**Fig. 8**). Thus, the ability of PTX3 to interact with the two regulators C4BP and CFH may limit excessive complement activation on apoptotic cells.

During pathological endothelial cell activation or following injury of the endothelial layer, the cells are retracted and the underlying ECM becomes exposed. Complement can be activated on the exposed subendothelial ECM *in vitro* [39]. In addition, as shown here, PTX3 binds to this ECM and by binding C1q it enhances the activation of the classical pathway (**Fig. 6** **and** **Fig. 7**), which through the amplification loop could also activate the alternative pathway. However, compared to host cells that are well protected from complement attack by membrane-bound regulators, the ECM does not have endogenous complement regulators, except for CD55 and CD59 released by endothelial cells [40], and mainly relies on soluble regulators that bind from plasma. Even though C4BP binds to the ECM without PTX3, PTX3 provides additional binding sites and enhances the binding of C4BP to the ECM. C4BP is able to regulate complement when bound on the ECM and/or to PTX3. Thus, PTX3 directs the complement inhibitory activity of C4BP to sites of enhanced classical pathway activation (**Fig. 7**). Importantly, under our assay conditions no competition between CFH and C4BP for PTX3 binding was observed, indicating that the two molecules could act simultaneously to control the alternative and the classical pathways, respectively. Thus, while complement activation is enhanced in the presence of PTX3, the generation of inflammatory C5a and the C5b-9 terminal complex, which could further activate or damage the endothelium, is not enhanced.

Although the concentration of PTX3 is relatively low in plasma, reaching up to 0.8 µg/ml concentration under inflammatory or infection conditions [4], it is produced locally by several cell types and can also be released by inflammatory cells such as neutrophils that have considerable amounts of PTX3 stored in specific granules [41]. Thus, PTX3 can reach potentially high local concentrations. PTX3 is produced by activated endothelial cells upon proinflammatory stimuli [1], [42] and as shown here, PTX3 released by the endothelial cells binds to the underlying ECM. Fibroblasts also produce PTX3 [43] which could similarly bind locally to the ECM. In addition, the synovial fluid from patients with rheumatoid arthritis contains strongly enhanced PTX3 levels, and synovial cells show a strong immunostaining for PTX3 [44]. The complement system will gain access to exposed ECM components during tissue injury, and upon activation on ECM it could further enhance inflammation and tissue damage. Exogenous PTX3 bound to both endothelial cell- and fibroblast-derived matrices *in vitro* (**Figs. 6** **and** **7**) and recruited functionally active C4BP. Thus, the interaction of PTX3 with the complement system to limit excessive complement activation may play a role under pathological conditions when the ECM is exposed during tissue injury, such as endothelial damage in hemolytic uremic syndrome or cartilage matrix exposure and degradation in rheumatoid arthritis [42], [44]–[46].

The short pentraxin and acute phase protein CRP has been shown to bind on one hand to C1q as a complement activator and on the other hand to C4BP and CFH as complement inhibitors [17], [19], [20]. As shown here, this capacity is shared by PTX3, which also binds C1q, C4BP and CFH. Thus, similar to CRP, PTX3 functions as a soluble pattern recognition molecule that cooperates with the complement system in target recognition. However, whereas native CRP appears not to enhance the binding of C4BP and CFH to apoptotic cells [34], PTX3 does enhance the binding of both C4BP (**Fig. 8**) and CFH [27]. Simultaneous binding of initiators (e.g., C1q, L-ficolin) and inhibitors (C4BP, CFH) of complement apparently ensures a targeted and limited complement activation that generates opsonins in a non-inflammatory way [23]. This appears as a mechanism shared by several endogenous host ligands, such as fibromodulin, osteoadherin, prion proteins, apoptotic and necrotic cells [23], [34], [45]–[47].

These results suggest that PTX3 and CRP can mediate similar functions, such as a regulated opsonization of target surfaces like necrotic/apoptotic cells or pathogens, via their complex interactions with the complement system [12], [13], [17]–[20], [25]–[27], [29], [36], [38], [48], [49]. A cooperation between pentraxins and complement regulators on endogenous ligands and damaged host cells is important to avoid the inflammatory and lytic effects of complement [15]. However, the site of action for the two proteins is likely different: while CRP acts in a systemic manner as an acute phase protein, PTX3 is more likely to act locally upon infection or inflammatory stimuli, e.g. on injured endothelium. In conclusion, our data indicate that PTX3 is capable of targeting functionally active C4BP to sites of tissue injury, thus limiting complement-mediated inflammation.
